# Designer's approach for scene selection in tests of preference and restoration along a continuum of natural to manmade environments

**DOI:** 10.3389/fpsyg.2015.01228

**Published:** 2015-08-19

**Authors:** MaryCarol R. Hunter, Ali Askarinejad

**Affiliations:** ^1^School of Natural Resources and Environment, University of MichiganAnn Arbor, MI, USA; ^2^Taubman College of Architecture and Urban Planning, University of MichiganAnn Arbor, MI, USA

**Keywords:** preference, restoration, well-being, environmental structure, evidence-based design, restorative urban spaces, psychological ecosystem services, design aesthetics

## Abstract

It is well-established that the experience of nature produces an array of positive benefits to mental well-being. Much less is known about the specific attributes of green space which produce these effects. In the absence of translational research that links theory with application, it is challenging to design urban green space for its greatest restorative potential. This translational research provides a method for identifying which specific physical attributes of an environmental setting are most likely to influence preference and restoration responses. Attribute identification was based on a triangulation process invoking environmental psychology and aesthetics theories, principles of design founded in mathematics and aesthetics, and empirical research on the role of specific physical attributes of the environment in preference or restoration responses. From this integration emerged a list of physical attributes defining aspects of spatial structure and environmental content found to be most relevant to the perceptions involved with preference and restoration. The physical attribute list offers a starting point for deciphering which scene stimuli dominate or collaborate in preference and restoration responses. To support this, functional definitions and metrics—efficient methods for attribute quantification are presented. Use of these research products and the process for defining place-based metrics can provide (a) greater control in the selection and interpretation of the scenes/images used in tests of preference and restoration and (b) an expanded evidence base for well-being designers of the built environment.

## Introduction

Over the past 50 years, environmental psychology research has made predictions about the mechanisms and outcomes of human response to the environment. Empirical research has shown repeatedly that people prefer and are restored by environments with nature or nature elements in evidence (reviewed in Velarde et al., 2007; Bratman et al., 2012; Ryan et al., 2014). Much less is known about the specific attributes of green space which produce these effects. In the absence of translational research that links theory with application, it is challenging to design urban green space for its greatest restorative potential. This paper describes an approach for defining which physical components, spatial characteristics, and collective arrangements of nature best meet theoretical predictions about preference and restoration in outdoor environments.

There has been some research on the impact of multiple physical attributes on preference or restoration. The importance of combinatorial effects in the environment has been noted by Berlyne (1970) who proposed that environmental scenes include collative properties, characteristics that cause a viewer to pay attention–Engagement, investigate further–Information Gathering, and compare. He predicted that when all of the collative properties occur in moderation, the viewer will perceive the setting as more beautiful. Ulrich (1983) offered support for this premise in his review of empirical work on aesthetic preference for specific visual properties of environment. With this data he considered the role of eight attributes (all but two were structure attributes) and was able to infer which character states in which combinations should be most preferred. Ryan et al. (2014) took a similar approach and were able to infer which specific physical attributes (design parameters) support Biophilia, people's need to connect with the natural world. Ewing and Handy (2009) used a different approach to decipher urban environmental complexity. Using an expert's panel, physical characteristics of streets, and their edges that best represented eight urban design properties (e.g., imageability and transparency) were identified.

Such research outcomes can serve as the premise of evidence-based design, a process that translates research outcomes for creation of the built environment, be that new development, restoration, or conservation (Hunter and Hunter, 2008). Here is an example of how the process could work for the design of restorative spaces. Ulrich (1983) noted that humans perceive nature stimuli as visual arrays more than individual objects and that we are strongly engaged by information that includes redundant elements, groupings of elements, patterns established by homogeneous texture, and properties that bring continuity to separated or dissimilar elements. Here, is a typical urban example that satisfies all criteria and does so with nature elements. Consider the view down a street that is symmetrically flanked by street trees planted at regular intervals. Since the early sixteenth century, roadways and promenades have used this structural format (an *allée*) to great effect (Pradines, 2012). Gestalt theory predicts that the structural/organizational properties of the whole will influence aesthetic preference. With an allée, trees provide the main lines of sight, guiding the eye toward a chosen focal point, often a vista (defined by the sky, horizon line, and land beyond), a perspective that opens into infinity. The content and structural form of this oft repeated form is known to be engaging, and even transcendent, depending on the chosen focal point (e.g., the sky). The success of the allée can be explained in terms of longstanding design principles as well as theories about survival, success, and aesthetic pleasure.

In this time of global urban densification, designers of the built environment are in need of a broader evidence base about which content and structure (spatial configuration) attributes offer the best chance to support restoration and well-being. Currently, the most common solution is to emulate natural scene aesthetics. This is not sufficiently prescriptive to meet the challenge of shrinking available space for greening. Designers are already well aware that preferred urban spaces are those with plants and water, attractive framing of good views, screening of buildings and roads, and presence of the disappearing curved path into vegetation, these being based on design principles that have been functional for centuries if not millennia (Jellicoe, 1995), and they continue to be taught today. What is most needed is better information about which physical components of a scene, acting alone or contextually, are involved in preference and restoration responses to urban nature.

Landscape designers use longstanding design principles to support the creation of settings that meet aesthetic, environmental and land use goals of a project. They also routinely apply research outcomes from the fields of ecology and engineering to meet environmental regulations and sustainability goals. The embrace of environmental psychology research outcomes has been limited for two reasons: there are few directives beyond “green is better, manmade is bad” that transcend what is already provided by design principles and aesthetics theory. Here, we describe an approach to help crack that generalization with a list of measureable, physical attributes (*aka* design components) that can be tested for their ability to support human well-being and better provide psychological ecosystems services in urban areas.

This study describes an approach to identify specific and measureable physical attributes of an environmental setting that are most likely to influence preference and restoration responses. The approach is practical, efficient, and founded in theory and existing empirical research. The process begins with a search for commonly invoked properties of spatial structure and content in theory predictions from the fields of environmental psychology, aesthetics, and design. This is followed by discovery process to identify specific physical attributes of the environment that fulfill four exacting criteria, including the ability to be measured.

## Methods

The sequential process described below allowed us to weave interdisciplinary considerations from several fields to identify physical attributes of nature most likely to generate preference and restoration responses.

(1) Set the criteria for which theories to consider. We chose among theories from the fields of environmental psychology and environmental aesthetics that include predictions about human preference for nature and/or restoration from exposure to it. We also considered design principles, historically-embraced in the fields of art and design. These principles are founded in the generation of form and space thought to be in keeping with the harmony of the universe and human aesthetic response. Design principles offer rich insight about how spatial configurations of content are perceived and which configurations are most engaging or restorative.

(2) Identify structure-content properties based on commonly held predictions about preference and restoration among the selected theories. Theory predictions were sorted into topically-related categories (structure-content properties), each of which was named with an encompassing descriptor.

(3) Identify specific physical traits (attributes) most likely to be involved in the expression of shared predictors of preference and restoration (structure-content properties). Inclusion of a physical attribute required that four criteria be satisfied: (1) alignment with theory-based shared properties; (2) alignment with formal design principles and/or empirical evidence or logical support for the relation of an attribute to preference or restoration; (3) ability to be measured objectively, and (4) capacity to be readily constructed, controlled or conserved by designers charged with the task of creating preferred and restorative outdoor spaces.

Our discovery process of appropriate attributes made use of the design process, an approach to investigation and product development used universally by landscape architects and architects. The design process works iteratively among the following tasks: gathering information (including evidence-base offerings from research where available), analyzing information relative to the project goal, and design creation using logic, intuition, creativity, visual communication, and testing for intended function.

(4) Ensure that attributes can be reliably measured. Since attributes are to serve as a set of testable hypotheses about which specific physical attributes of outdoor settings contribute to preference or restoration responses, there must be a measurement method that is readily used by other researchers. The metrics of each attribute were defined and adjusted throughout the discovery process until they were fully functional–meaning that the character states were easily understood, efficiently measured, and collectively encompassed the range of possible outcomes.

Throughout the process of attribute definition, one discussion was recurrent—how to readily measure 3D features of the outdoor environment using 2D representation (e.g., photos). This focus also helped avoid the problem of picking physical attributes that would be impossible to assess from images owing to unfounded assumptions (e.g., about what lies beyond the frame of view). Examples of attributes with good objectivity include horizon line position, perspective view, and the ever popular % green vegetation.

A key part of the iterative design process was testing the clarity of attribute metrics. This was done with 400 images collected from participants in another research project who had been charged with taking pictures of scenes that produced a positive reaction during a nature experience. Since compositional quality (e.g., presence of a focal point, balance, or unity) can make all the difference in response of a test subject to a scene, the use of this image source ensured that the method of scene analysis that was not dependent on the quality of the photo.

Attribute definitions and metrics (verbal and graphical) were adjusted and retested using images never before seen by scorers until consensus was reached. Throughout this process, three trained scorers worked independently then compared results. Where discrepancy occurred, a 4th scorer was consulted, followed by group discussion and decision about what adjustment to make.

For most attributes, convergence was 100% by the end of the definition/metric building process. For several attributes, convergence was not achieved 2–4% of the time and group discussion and decision was still required; use of an outside scorer was valuable in such cases.

For attributes defined terms of area, the perimeter was outlined in Photoshop and data were processed in Grasshopper with a program developed to efficiently make area calculations. Area occupied by an attribute was calculated as the percent of an image's total area, allowing measurements to be calibrated for comparison.

## Results

### Theories chosen for consideration

Theories about human response to nature predict that a preferred or beneficial setting is one that offers protection, supports resource acquisition and, if survival-reproduction needs are met, provides opportunities for adventure or transcendence. Predictions from the following theories were used as the basis for identifying physical attributes of a scene or image that activate preference or restoration responses.

The first three theories are evolutionary in essence, focusing preference responses to environmental stimuli that are innate, an outcome of natural selection for survival and fitness. Evolutionary aspects of environmental preference posit that scenes offering the resources and opportunities necessary for success will elicit a positive aesthetic reaction (e.g., pleasurable) and have come to be preferred on that basis. Biophilia (BT) predicts the urge to affiliate with the natural world and its diversity, particularly, the diversity of landscapes, habitats, and species (Wilson, 1984). Habitat/Prospect-Refuge theory (PRT) predicts that human experience of pleasure and satisfaction is associated with landscapes that meet biological needs for survival and success. Even in the absence of imminent danger or need, the positive aesthetic response to valued or formerly valued landscapes will occur (Appleton, 1975). More specifically, Prospect-Refuge predicts a preference for places that offer outlook, enclosure, and aesthetic pleasure. Savanna theory (ST), a corollary of Prospect-Refuge theory, predicts a preference for places with the spatial form of the savanna habitat where critical phases of human evolution occurred. Savanna form is typified by open grassland for prospect and intermittent climbable trees for refuge (Orians and Heerwagen, 1992).

The next three theories variably address the roles of affect and cognition in landscape preference and restoration without precluding innate response. Stress Recovery theory (SRT) (Ulrich, 1983; Ulrich et al., 1991) predicts an improvement in emotional and physiological states in response to a non-threatening nature experience, all of this mediated by the initial affective state of the individual. A preference response to a setting is initiated by an affective aesthetic reaction which is in part hardwired, the outcome of natural selection for survival and success. More specifically, Ulrich (1983) predicts that natural scene preferences will be related to aspects of a setting's visual properties in terms of structure, organization, and general content classes such as water or man-made components that support survival and success. Environmental Information Processing theory (EIPT) predicts preference for landscapes that facilitate information gathering and support the capacity to plan for the purpose of survival, success, and further exploration. Preference is based on information processing of what is directly observed of a landscape's coherence and complexity along with what is inferred about its legibility and mystery (Kaplan, 1987). Attention Restoration theory (ART) predicts that the experience of nature restores the capacity for directed attention when nature is configured to create the sense of being away from everyday thoughts and concerns; to provide fascination defined as effortless attention; to offer sufficient content and structure to occupy the mind long enough for one's directed attention to rest; and to have a compatibility between one's purposes and what the environment offers. Interpretation of a scene can involve desires, memory, and experience, although innate response can transcend the personal details of why a setting is suitable (Kaplan and Kaplan, 1989; Kaplan, 1995).

The next theories focus on the role of aesthetic response in landscape preference and restoration responses. These offer a useful stepping stone for operationalizing environmental psychology predictions because they are more specifically focused on physical elements of landscape design. Environmental Aesthetics theory (EA) joins physical and emotional criteria used in the world of art to define aesthetic response in terms of meaningful outcomes ranging from survival to transcendence (Carlson, 2015). Environmental Aesthetics via Urban Design (EA-Urban) makes predictions about the attributes of a successful city. The emphasis is on built structures, nature elements being left primarily as modifiers. Nonetheless, understanding which forms and spatial configurations are preferred and restoring is of interest because most people live in urban settings worldwide. Where there is limited space for nature in the city, it is critical to know which built content, configured in what ways, including how much nature will be preferred and most supportive of restoration. The works of Lynch and Ewing are useful here because they deal with aspects of design that transcend which materials are employed–natural, man-made or man-made emulating nature. Lynch's (1960) *Cognitive Mapping* identifies and makes predictions about five elements that support wayfinding which make the landscape comprehensible and useful. Ewing et al. (2006) proposed that *Walkability* could be better integrated with city design by evaluating the design in terms of nine urban design qualities, many of which were put forth by Lynch (1960).

A Pattern Language*: Towns, Buildings, Construction* (Alexander et al., 1977) is guide to urban design, organized as a hierarchical network of interrelated design solutions that lead to desirable built spaces. This book offers a set of hypotheses in the form of 253 “patterns” each of which describes a problem about the spatial configuration and context of communities, streets, or buildings that influence the quality of life. Each problem is paired with a set of design solutions to improve the aesthetic experience and human well-being. The validity of both problem and solution is based on the history of successful built spaces, empirical research outcomes, and the design and construction experience of the authors. The book is aimed at urban planners and architects but is included here because both problem and solution often disclose specific physical attributes of three dimensional space that influence preference and restoration.

Scenic and Landscape Aesthetics theories (S-LA) are distinct from Environmental Aesthetics in their approach for evaluating the scenic quality of landscapes which often includes valuation of ecological function (USDA-FS, 1995). The goal is to produce criteria for landscape conservation and development that provides, saves, and frames beautiful views as the viewer moves through a larger scaled landscape such as a public park or along a highway. It is included here as it is based on psychophysical criteria about preferred views (scenic beauty) for viewshed management of large scale landscapes. And it employs measurable landscape characteristics (Daniel and Boster, 1976).

Design Theory–Formalized Principles (DP) Formal design principles have emerged over millennia. Many principles are based in mathematics and the long held belief that numerical relationships manifest the harmonic structure of the universe (Ching, 2007) and, by extension, will be preferred and in some way restorative. Design principles are the vocabulary for creating an object, a setting, or a series of settings where form and space are ordered to bring unity, balance, and a spatial or temporal hierarchy to the whole. These characteristics play a key role in aesthetic response. Visual engagement and interpretation is founded on the arrangement of lines, forms, and their sensory attributes (like color and texture) in terms of proportion, scale, and ordering principles such as symmetry and rhythm. The body of knowledge summarized in design principles continues to be used worldwide by design professionals and artists.

Many formal design principles like the golden mean and the location of a horizon line) are foundational for the digital field of visual aesthetics (e.g., Bhattacharya et al., 2010). It is not a surprise that many of the design principles for preferred arrangement of scene elements are coherent with what is predicted to be desirable by psychophysical models. It has been recognized by researchers that some of the traditional aesthetic domains may be derived from more basic functions of survival and success, such that environmental aesthetics is not a special case of aesthetics but a reflection of its broad and pervasive utility (e.g., Kaplan, 1987).

### Identity of structure-content properties about preference and restoration

A comparison of predictions from all theories revealed shared structure-content properties of greatest relevance to preference and restoration responses (Table 1). Each structure-content property was named with an encompassing descriptor for a set of topically related theory predictions. Only shared properties supported by the predictions of at least 3 of the 10 eligible theories were included for further study. Although theories share predictions (i.e., are to some degree co-correlated), it is valuable to consider the context from which the prediction emerges in order to (a) evaluate the relative importance of structure-content properties for preference and restoration, and (b) more ably identify which specific physical attributes are in play.

**Table 1:** **Identification of shared structure-content properties predicted to be important in preference and restoration responses (across) by key theories from environmental psychology and environmental aesthetics (down)**.

	**Naturalness**	**Complexity**	**Structural coherence**	**structural form**	**depth cues**	**Openness**	**Information gathering support**	**Access**	**Safety**	**Engagement**
Biophilia theory (BT)	X	X								
Habitat: prospect-refuge theory (PRT)	X				X	X	X	X	X	
Savanna theory (ST)	X			X	X	X	X	X	X	
Stress recovery theory (SRT)	X	X	X	X	X		X	X	X	X
Environmental information processing theory (EIPT)	X	X	X	X	X			X	X	X
Attention restoration theory (ART)	X								X	X
Environmental aesthetics (EA)	X	X	X	X		X	X		X	X
Environ. aesthetics via urban design (EA/Urban)		X	X	X	X	X	X	X	X	X
Scenic or landscape aesthetics (S/LA)	X	X	X	X	X	X		X	X	X
Design principles (DP)	X	X	X	X	X	X	X	X	X	X
Frequency of concurrence	9	7	6	7	7	6	6	7	9	7

Naturalness is defined as any type of nature content—the presence of biota, land, water, or sky. Nine of 10 relevant theories predict naturalness as foundational to preference for and/or restoration from the landscape. Design principles, rooted in observation and analysis of the natural world, recommend imitation of nature's rules of form and changeability to create a satisfying outcome. By contrast, environmental aesthetics theory via urban design focuses on human built forms in the landscape (EA/Urban: Ewing et al., 2006).

Complexity is defined as information richness of a scene deriving from diversity in its physical structure and physical content. The elements of complexity emerge from variety in line types, forms, textures, or color (DP). Seven theories predict complexity to be related to preference. Biophilia subsumes both structure and content into the term resource variation which refers to the ecological capacity of the environment to provide food, protection, and space for activities important to success (BT: Wilson, 1984). Complexity is predicted as preferred, particularly at moderate levels (SRT: Ulrich, 1983), because it heightens the potential for exploration (EA: Berlyne, 1970; EIPT: Kaplan, 1987). Urban design aesthetics predicts that a walkable city includes the complexity that emerges from variety in building type and spatial grouping, architectural diversity and ornamentation, landscape elements, street furniture, signage, and human activity (EA/Urban: Ewing et al., 2006). At the landscape level, shape complexity and landscape diversity are predicted to be related to preference (S/LA: Schirpke et al., 2013).

Structural Coherence is the degree of unity and visual order often achieved through patterning or linkage of scene components (e.g., a linear succession of tree trunks or canopies. Six theories consider structural coherence of importance to preference or restoration. It is founded on symmetries, repeated elements, homogeneous textures, content or color patterns that bring balance and unity, and the presence of a focal point in a scene (DP; EA: Berlyne, 1971; SRT: Ulrich, 1983; EIPT: Kaplan and Kaplan, 1982). Massing Structural coherence also comes from the balance of repeated elements along an axis (symmetry) and massing of like elements to create a line of visual interest that activates a scene (DP). Structural coherence is also influenced by consistency and complementarity in the scale, character, and arrangement of physical elements like buildings or associated nature elements (EA/Urban: Ewing et al., 2006) and by correspondence between land use and natural conditions of an area (S/LA: Tveit et al., 2006).

Structural Form is present as gestalt, the scene with structural form appears as an organized whole that is more than the sum of its parts (SRT: Ulrich, 1977; EIPT: Kaplan, 1984) This happens with habitat types, like savanna (ST: Orians and Heerwagen, 1992), and biomes, like forest (S/LA: Han, 2007). The gestalt of a city can be defined by its imageability, having a form that is instantly recognizable (EA/Urban: Lynch, 1960). Seven theories predict structural form as important to preference and restoration. It is foundational to the aesthetic response called beauty (EA: Shapshay, 2013). Structural form involves organizing principles like style (e.g., fine textured, highly geometric, DP), emerging from the configuration of lines and planes that can be dominated by curves, straight lines, sharp angles, or some mixture of these.

Depth Cues help us understand the proportional relation and size of objects in a scene. Seven theories predict preference for scenes that support depth perception. A scene with sufficient depth to evaluate the presence of resources and danger is predicted as more preferred (PRT: Appleton, 1975; ST: Orians and Heerwagen, 1992), as are landscapes dominated by the experience of moderate depth (SRT: Ulrich, 1983). A key outcome of successful information gathering is the ability to go deeper into a scene, implying that depth perception is critical to preference development (EIPT: Kaplan et al., 1972). Depth cues reveal proportional relationships between size and distance. Object size can also be inferred by proportional sizing or *human scaling*—using the relative size of known objects or one's own body as the metric for size and distance of what lays beyond (DP). This approach to proportional sizing is enhanced when the arrangement of lines and forms produce a perspective view as happens overtly in the streets of most urban settings (EA-Urban). At the landscape scale, proportional sizing is based on the relative size of objects in the fore, mid, and background of a scene (S-LA: Schirpke et al., 2013).

Openness is defined as a position along a continuum from physical or visual spaciousness to full enclosure. Six theories predict that degree of openness to be important in preference and restoration responses. Scenes with sufficient openness to evaluate the presence of resources and danger are predicted as preferred (PRT: Appleton, 1975; ST: Orians and Heerwagen, 1992). Openness is a key consideration of designers and urban planners who measure it in terms of volumetric proportion, often using human scale to dimension outdoor spaces that serve well-being (e.g., EA/Urban: Ewing et al., 2006; DP: Alexander et al., 1977). Landscape planners make similar considerations about the form of openness when designing for recreation, habitat restoration, and recovery from disturbance (S/LA: Tveit et al., 2006). Openness has also been defined as nature content arranged with a spatial structure that brings a sense of being surrounded by nature or a sense of nature's boundlessness (EA: Hepburn, 1996). This definition bears directly on the goal of landscape designers charged with creating a sense of the natural world within a dense urban area.

Information Gathering Support includes features of the environment that support the ability to learn more about a setting, often by moving deeper into it. Six theories predict that such support in relevant to preference and restoration responses. Examples include the presence of a physical vantage point to see what is beyond (PRT: Appleton, 1975; ST: Orians and Heerwagen, 1992), a focal point (SRT: Ulrich, 1983), a guiding line to directs visual attention (EA: Berlyne, 1970), plus the degree of visual transparency and the presence of wayfinding tools in the scene (EA/Urban: Ewing et al., 2006). Information gathering is supported by the application of design principles (DP) such the presence of perspective, organizational symmetry of nature–like paired trees along the edge of something, and the configuration of complexity with an organizational spatial hierarchy–like a canopy, understory, and groundcover of vegetation.

Access is about having sufficient and readily understood information that is useful for navigation through an environment. It is a purpose-driven associate of the structure-content property called information gathering support. Seven theories make predictions about features of access that are relevant to preference and restoration responses. Features include having a safe place from which to plan a route (PRT: Appleton, 1975; ST: Orians and Heerwagen, 1992), and having a type of ground surface suitable for navigation (SRT: Ulrich, 1983). Comprehensible movement routes are important in unfamiliar urban areas for wayfinding (EA/Urban: Lynch, 1960) while an obscured view with limited visual access can lead to unpleasant surprises (EIPT: Kaplan and Kaplan, 1989). Path-space relationships and circulation design commonly apply an array of design principles with the goal of maintaining perceptual clarity while providing interest and beauty (DL: authors; Ching, 2007).

Safety is based the presence of environmental form and features that offer protection (or not), especially while gathering information. Nine theories make predictions about features of safety relevant to preference or restoration responses. In terms of environmental structure, safety is typically attributed to places that provide a sense of boundary, access to refuge, or an escape route along with observation point(s) to see what is beyond (PRT: Appleton, 1975; EA-Urban: Chiang et al., 2014; ST: Orians and Heerwagen, 1992). Features of such places include attributes that bring legibility (EIPT: Kaplan, 1987) and the absence of threat (SRT: Ulrich, 1983; Ulrich, ART: Kaplan and Kaplan, 1989). In urban settings, a balance between perceived safety and the degree of naturalness can influence legal regulation (EA: Pearlman, 1988) as well as preference and the capacity for mental restoration (S/LA: Schroeder and Anderson, 1984). The ongoing challenge is to provide safety in balance with positive aesthetic qualities at all landscape scales (e.g., S/LA: Fathi and Masnavi, 2014). Vigilance with safety-aesthetics considerations during design and construction of the built environment, is a hallmark of human settlements (DL: Hill, 1996), for example, the formulas for depth and height of stairs inside or out.

Engagement is based on the presence of something physical that holds the attention. For some this includes the concept of imageability which happens when specific physical elements and their arrangement capture attention, evoke feelings, making landscapes distinguishable, and memorable. (EA/Urban: Ewing et al., 2006; S/LA: Tveit et al., 2006). Seven theories predict preference or restoration from scenes that offer engagement. Engagement can arise in the presence of an attention-getter (SRT: Ulrich, 1983) or from content or structural form that creates mystery or ignites the imagination (EA: Godlovitch, 1994; Brady, 1998). Engagement influences preference especially through ephemera, often the result of weather changes (ART: Kaplan, 1995; S/LA: Tveit et al., 2006). Mystery, the promise of new things to explore if you move further into the landscape, is a consistent predictor of engagement and landscape preference (EIPT and S/LA: Kaplan et al., 1989). Design principles collectively aim for aesthetic engagement, regardless of other goals such as function or safety (DP: authors). Because the engagement attribute is so rich in meaning, we divide it into two categories based on the predictability of physical form that elicits the engagement: (1) engagement from predictable content, includes ever present and fixed objects like a landmark and predictable phenomena such as sunset or fall color, and (2) engagement from unpredictable content, includes ephemera such as rainbows and objects (like foliage) with the capacity for unpredictable movement and interaction with light and water.

Other Considerations. Features of four theories overtly predict the importance of personal meaning to preference and restoration response. This shared property, Highly Meaningful (not shown on Table 1), did not move forward to the next phase of work because its measurement is far less relatable to specific physical structure and content features of the environment and is too subjective for direct measurement. Additional information would have to be gathered from subjects for interpretation, such as place-based contributions to preference and restoration (Wilkie and Stavridou, 2013). Theory predictions from the Highly Meaningful category included “being away” and “compatibility” from Attention Restoration theory, “threat” from Environmental Aesthetics theory, and “stewardship”, “disturbance”, “tidiness”, and “historicity” from Scenic or Landscape Aesthetics theory.

### Identity of physical attributes associated with structure-content properties

The following attribute list articulates concrete predictions about which specific physical structures and content of a landscape are most relevant to preference and restoration. Attribute definition is the final critical step in operationalizing theory predictions.

We identified 62 physical attributes likely to influence preference and restoration. Each of these fulfilled the four requisite criteria: (1) directly manifests a key aspect of at least one theory-derived structure-content property (reported in Tables 2–4), (2) could be defined with a standard for measurement, (3) had empirical evidence from research or design logic supporting its relevance to preference or restoration, and (4) could be constructed, controlled or conserved during the task of creating preferred and restorative outdoor spaces.

**Table 2:** **Identity of physical structure attributes that manifest a key aspect of at least one structure-content property**.

**ID#**	**Structure attributes**	**Naturalness**	**Complexity**	**Structural coherence**	**Structural form**	**Depth cues**	**Openness**	**Info gatheringsupport**	**Access**	**Safety**	**Engagement (predictable)**	**Engagement (unpredictable)**	**Alexander pattern number^a^**
1	Horizon Line position			1		1		1					
2	Skyline position					1							
3	Perspective Type			1	1	1		1	1				
4	Scenography type				1	1	1						114
5	Building distribution				1		1	1	1				53
6	Canyon form				1		1						
7	Water expanse				1	1			1				25
8	Habitat type				1								
9	Trunk position-nearby					1				1			171
10	Framing					1		1			1		134, 239
11	Framing tree count	1		1		1							
12	Viewer in shade				1			1		1			135
13	People proximity					1		1				H	
14	Built surfaces to move			1				1	1				52, 129
15	Visual access to path							1	1				
16	Direct Access to Path			1				1	1				
17	Cover type on circulation			1		1			1				
18	Circulation boundary		1	1				1					
19	Skyline width in frame						1						
20	Skyline geometry		1		1								116
21	Skyline max undulation		1										
22	Skyline vibrancy—proportion	1	1	1								E, F	
23	Skyline vibrancy—length												

a *Engagement code and Alexander Pattern number key with Table 3*.

**Table 3:** **Identity of physical content attributes that manifest a key aspect of at least one structure-content property**.

**ID#**	**Content attributes**	**Naturalness**	**Complexity**	**Structural coherence**	**Structural form**	**depth cues**	**Openness**	**Info gathering support**	**Access**	**Safety**	**Engagement (predictable)**	**Engagement (unpredictable)**	**Alexander pattern number^a^**
24	Natural phenomena	1									A-D	E–H	
25	Water form	1	1	1	1		1				D	E–H	64
26	Distinct shadows		1	1		1		1				E	135
27	Focal objects			1				1			1		126
28	Wayfinding objects							1	1				
29	Lighting							1		1		E, F	252
30	Seating							1		1			241
31	Windows		1			1		1		1		G	57, 164
32	Vehicles					1				1		H	52
33	Animal presence	1				1				1	1	H	74
34	People presence					1				1	1	H	100
35	Portrait										1		

*Engagement code key: 1-A, diurnal shifts; 1-B, tidal shifts; 1-C, seasonal changes; 1-D, predictable movement; 1-E, wind and light induced change; 1-F, sparkle, visual vibrancy; 1-G, reflections; 1-H, unpredictable movement*.

a *Alexander Pattern number key: #25, access to water; #51, green streets; #52, network of paths and car; #53, main gateways; #64, pools and streams; #57, children in the city; #74, animals; #100, pedestrian street; #114, hierarchy of open space; #116, cascade of roofs; #126, something roughly in the middle; #129, common areas at the heart; #134, Zen view; #135, tapestry of light and dark; #164, street windows; #171, tree places; #238, filtered light; #239, small panes; #241, seat spots; #252, pools of light (Alexander et al., 1977)*.

**Table 4:** **Identity of physical landscape attributes that manifest a key aspect of at least one structure-content property**.

**ID#**	**Landscape attributes**	**Naturalness**	**Complexity**	**Structural coherence**	**Structural form**	**Depth cues**	**Openness**	**Info gathering support**	**Access**	**Safety**	**Engagement (predictable)**	**Engagement (unpredictable)**	**Alexander pattern number^a^**
60	**NATURE**												
→	Sky veiled (36), Sky open (43), Sky total (49)	1				1	1				A	D–F	
→	Water veiled (37), Water open (44), Water total (50)	1	1	1			1				B	E	
→	Earth veiled (38), Earth open (45), Earth total (51)	1					1		1				
42	Veiling vegetation	1	1	1		1						D	238
52	Non-veiling vegetation												
53	Vegetation total	1	1	1	1	1					C	D, F	
57	Vegetation Canopy												171
58	Vegetation Understory												
59	Vegetation groundcover												51
61	**MANMADE**												
→	Built structures veiled (39), Built structures Open (46), Built structures Total (54)		1		1	1					1		
→	Built ground veiled (40), Built ground Open (47), Built ground total (55)					1	1		1	1			
62	**OTHER**												
→	Other veiled (41), Open other (48), Open total (56)					1							

a *Engagement code and Alexander Pattern key with Table 3*.

The attributes fell into three design categories—structure attributes that focus on spatial configuration, content attributes that address the identity of non-landscape attributes, and landscape attributes that represent the natural and manmade content of a landscape; they are measured in terms of their coverage area in a scene. For better organization, attributes in each category are subdivided based on dominant commonalities of form or function where this exists. Attribute metrics include the easier-to-measure continuous variables like percent sky and bivariate variables like presence/absence of windows, to categorical variables that were defined to summarize a set of complex considerations.

The narrative that follows includes the definition, a metric, and the rationale for including each attribute. Figures provide a graphical version of definitions for complex spatial attributes. Note that the term Alexander Pattern refers to a pattern number from A Pattern Language (Alexander et al., 1977) that predicts or demonstrates the importance of an attribute to preference or restoration in urban settings.

#### Structure attributes (Table 2)

Design logic is the basis of support for each member of this group. Where available, empirical, and design research is mentioned.

The first eight attributes offer metrics for essential aspects of whole scene spatial structure.

**Figure 1:**
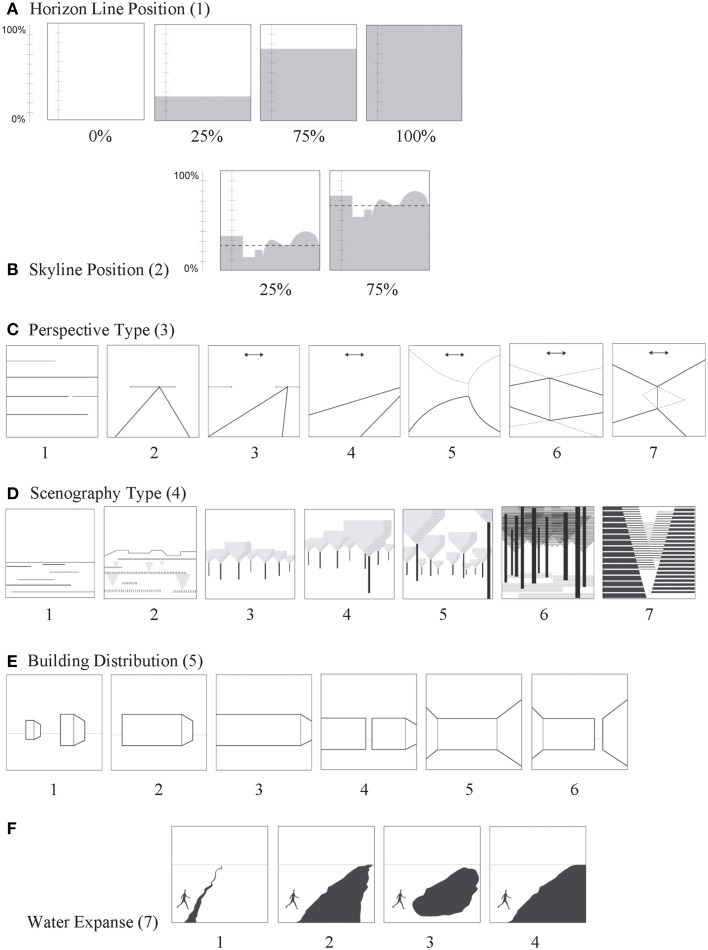
**Visualized definitions—character states of attributes with complex spatial definitions, part 1**. Attribute ID numbers given in parenthesis. Descriptions of numbered character states for attributes **(A–F)** are given in the section called Structure Attributes.

Horizon Line Position (1), where earth meets sky (seen or inferred position), is foundational for deciphering one's position and size relative to other objects in view. Horizon line position also contributes to the visual balance in a scene. Measured relative to the base of the frame's vertical axis; 0–100% (Figure 1A).

Skyline Position (2), the position of the habitat-sky interface, helps the viewer spatially interpret other structural features. It is the average position of a line of earthbound objects (natural or built) as they meet the sky, measured relative to the base of the frame's vertical axis; 0–100% (Figure 1B).

Perspective Type (3) is based on location of a scene's vanishing point(s) and is estimated with visual trajectory lines converging at the vanishing point. The vanishing point(s) is a located in response to the sculptural form of a scene and the information it provides about seeing or moving beyond obstacles. Perspective theory comes from the fact, first articulated in the early Renaissance that apparent size of an object decreases with increasing distance from the eye. Perspective types differ in focal point number and position(s) along the horizon line. In Figure 1C, 0, can't tell; 1, No vanishing point (e.g.,–elevation view, close up to a surface); 2, vanishing point in the center of horizon line (central third of frame); 3, vanishing point on right or left side of horizon line (right or left third of frame); 4, vanishing point out of the view frame but can be inferred; 5, Deflected: vanishing point is obscured by other objects so its likely position cannot be inferred (e.g., a trail that disappears around a bend); 6, two vanishing points outside the frame (e.g.,–a building seen on edge or a crossroads); 7, two vanishing points inside the frame but hidden (e.g.,–enclosed space like a courtyard or an outdoor room). Note that a deflected perspective (condition 5) was first shown to be preferred by Kaplan et al. (1972), who interpreted that a deflected was a source of mystery because of its promise for additional information by moving deeper into the scene. Design principles say that a perspective view offers scene information based on location and size of objects relative to one another, and a vanishing point at the horizon gives information about scene depth (Lebreton et al., 2014).

Scenography Type (4) is a gestalt variable that describes the proximity of a viewer to the landscape beyond in terms of its sculptural form and scene depth. Its character states represent a change from more to a less expansive view. This is akin to Alexander Pattern #114 “hierarchy of open space”. The key to measuring this variable is to consider the physical experience of scene form rather than its content *per se*. Figure 1D illustrates one example—the change in the spatial structure of one's view while approaching a distant woodland and gradually arriving: 0, can't decipher; 1, landscape extends from the viewer to a vista or a bird's eye view of an extended landscape, unbroken by nearby objects; 2, like condition 1 but with very nearby objects; 3, open area in scene's fore to mid-ground with taller objects/vegetation beyond; 4, like condition 3 plus one or a few trees/vertical objects near at hand but separate from the objects/vegetation beyond; 5, open area in the foreground with vegetation/objects that extend continuously into the distance; this type includes most urban street tree scenes; 6, embedded but with a view of what is beyond; 7, embedded with no useful view of what's beyond. Schirpke et al. (2013) found that landscape scenes with foreground elements got higher preference ratings for scenic beauty over those with mid, and far ground elements. Herzog and Bryce (2007) found that preferred focal lengths for prospecting were >100 feet compared to shorter ones less than 20 feet.

Building Distribution (5) signifies the configuration of building or building clusters as they influence a viewer's visual and physical access to what is beyond. In Figure 1E, 0, no buildings; 1, all buildings/clusters are distinct with many openings or inferred openings; in urban street perspectives, visual, or physical porosity is evident; 2, building/clusters block the majority of view beyond but are sufficiently open to infer what is beyond; 3, building/clusters completely block the view beyond but a way to move beyond can be inferred; 4, like condition 3 but with a passage way (able to detect light, vegetation or what is beyond); 5, building/clusters surround the space and views are blocked; 6, like condition 5 but with a passage way (able to detect light, vegetation, or what is beyond). Stamps (2005) reported that the impression of enclosure is related to the percent of a scene covered by surfaces that block vision and movement. Ewing et al. (2006) noted that transparency can be adjusted by the design characteristics of walls, windows, doors, fences, landscaping, and building placement. The access porosity speaks to Alexander Pattern #53 about the importance of gateway experience wherein visual or perceived boundaries are crossed at access points. Interestingly, the passage point itself helps maintain the perceived integrity of the boundary.

Canyon Form (6) is scored when the landform (natural or built) produces the enclosure effect of a canyon; No-Yes. This structural form has been a valuable resource owing to protection from temperature extremes through solar shielding and at nighttime conservation of heat (Levermore and Cheung, 2012).

Water Expanse (7) is based on physical or visual access across a water body; physical access is defined as the ability to cross the water by foot. Its role in well-being is addressed in Alexander Pattern #25 “access to water”. In Figure 1F, 1, crossable linear waterway (e.g., narrow stream or runnel); 2, water body not crossable and viewer can see its other side; 3, water body not crossable and its entire boundary can be seen; 4, water body not crossable and viewer can't see to its other side. Nature and urban scenes holding water bodies were more preferred and restorative than those without (White et al., 2010).

Habitat Type (8) can be measured in either or both of two ways depending on the image set. Habitat Type 8A is the natural ecological landscape type revealed at least somewhere in the scene. Each type is dominated by characteristic forms that allow identity at a glance: Types are: 0, can't tell; 1, forest; 2, grassland/prairie; 3, forest + grassland/prairie; 4, coastal/edge area of a water body (e.g., lakes, oceans, wide rivers); 5, savanna; 6, desert; 7, tundra; 8, urban/urbanized; 9, agricultural; 10, residential scenes with insufficient information about larger context and close up scenes with human scale structures. Habitat Type in the built environment (8B) emerges from design or management of natural elements. Emulation is founded on the structural form of natural habitat types. It is most often seen in gardens, parks, and greened portions of the built environment Types are: 0, no emulation; 1, forest form; 2, grassland/prairie form; 3, forest + grassland/prairie form (e.g., turf area abutting woodlot); 4, coastal/edge area of a water body (e.g., designed plantings around stormwater pond); 5, savanna-like (e.g., low grass and spaced out trees, a form found in many urban parks); 6, desert form; 7, tundra form; 8, garden form - stylized (or manmade form - stylized); 9, agricultural form (e.g., urban agriculture plots, raised beds); 10, garden form- not stylized.

The next five attributes address the presence of objects near to the viewer.

**Figure 2:**
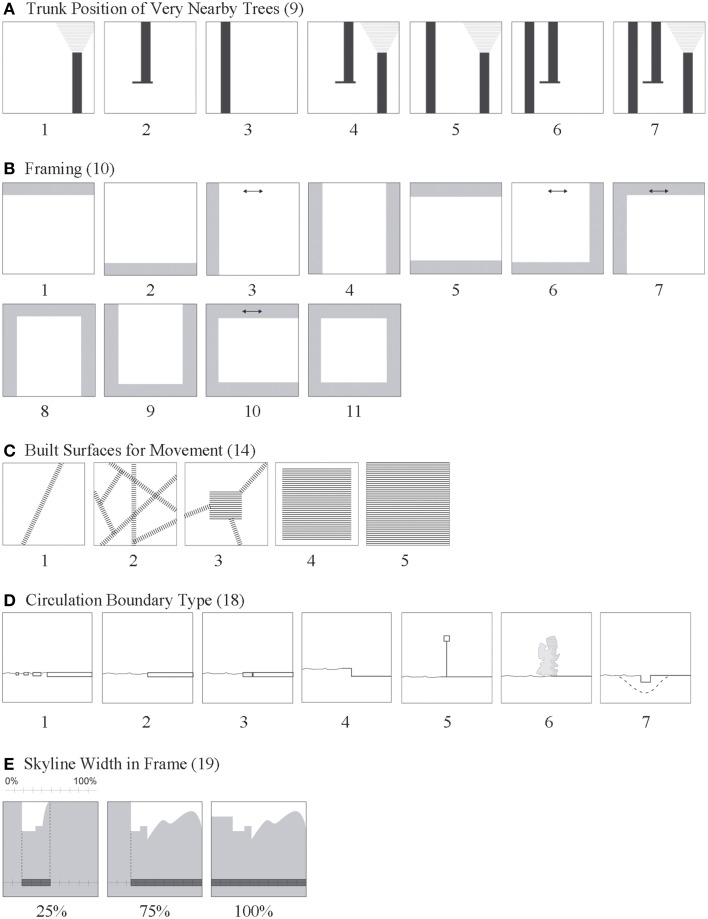
**Visualized definitions—character states of attributes with complex spatial definitions, part 2**. Attribute ID numbers given in parenthesis. Descriptions of numbered character states for attributes **(A–E)** are given in the section called Structure Attributes.

Trunk Position of Very Nearby Trees (9) is based on evaluation of trees that are close enough to produce a visceral sense of proximity and are distinctly spatially separated from non-nearby trees. This attribute is addressed in Alexander Pattern #171 which describes how nearby trees produce desired spatial structures that support well-being. Trunk position is scored relative to trunk intersection (or not) at the top and bottom edges of the scene. In Figure 2A, 0, no nearby tree trunks; 1, trunk emerges from bottom frame and ends within frame; 2, trunk emerges from ground plane within scene and continues past top of frame; 3, trunk runs from bottom to top of the scene; 4, 1+2; 5, 1+3; 6, 2+3; 7, 1+2+3. (In cases where Scenography attribute is rated as condition 5, 6, or 7, trunk position of only the nearest tree is scored.)

Framing (10) A framing object is very near to the viewer, it partially obscures what is beyond by having boundaries that extend beyond the image frame. There are 11 framing classes ranging from 1 to 4-sided framing, arranged in all possible edge configurations; 0 = no framing. See Figure 2B. Because framing acts to engage one's focus to what is beyond, it is routinely used by designers and artists to guide attention. Framing objects support information gathering and offer depth cues by serving as a foreground object for scaling (Schirpke et al., 2013). Ryan et al. (2014) suggest that mystery occurs when a scene's boundaries are partially obscured on one or preferably two edges; this may be particularly so when the framing object is foliage (Gimblett et al., 1985). Alexander Pattern #135 “Zen view” and 239 “small frames” say that framing objects limit visual access thereby preventing habituation to the view beyond.

Framing Tree Count (11) is the number of sides of a frame with trees as framing objects. Here, 0, 0; 1, 1; 2, 2; 3, 3; 4, 4. As tree count of framing trees goes up, there should be an increased sense of safety and visual linkage which brings a sense of continuity with what is beyond.

Viewer in Shade (12) happens when the vantage point is sheltered in shade which contrasts sharply to a bright scene beyond. The contrast brings greater visual emphasis to what lies beyond; No-Yes. This is akin to the Alexander Pattern #135 “tapestry of light and dark”. People Proximity (13) relates the position of people in a scene relative to the viewer's position. The human body functions as a scalar for depth perception (designers call this human scaling). Here, 0, no people, 1, people near, 2, people far, 3, people near and far.

Ability to move through the environment is essential for survival and well-being. The next five attributes concern transit corridors.

Built Surfaces for Movement (14) is defined by the type of the designated circulation system found in the scene, with type based on physical configuration. Alexander Patterns describe benefits of well-designed circulation networks (#52) and nodes (#129). In Figure 2C, 0, no circulation system; 1, single path; 2, network of paths; 3, path(s) with node(s), the node being a useful space, not simply an enlarged intersection; 4, free movement over broad space and can see its boundaries (e.g., plaza, patio); 5, free movement over broad space but can't see its boundaries; 6, movement surface is visible but can't read the circulation configuration (no associated graphic in Figure 2C). The character states of this variable describe in the ability of lines to direct and orient. Accessibility and provision of a view was also very important to preference in Scandinavian forests (Gundersen and Frivold, 2008). The issue of accessibility can be addressed with this metric by grouping outcomes as yes-no.

Visual Access to Path (15) occurs when the viewer can see a designated path or circulation surface; No-Yes.

Direct Access to Path (16) occurs when the viewer appears to be on a clearly designated path or circulation surface; No-Yes. Sense of depth is heightened when the viewer is on a path that moves into a scene as this provides a perspective view.

Dominant Cover Type on Circulation Surfaces (17) defines the surfacing material on designated circulation surfaces. Types include: 0, no circulation system; 1, sand or compacted soil; 2, mulch; 3, turf grass (mowed); 4, gravel; 5, tiled (tile, paver, flagstone, stone, cobblestone); 6, paved (asphalt/concrete); 7, tiled and paved; 8, wooden. Surface texture and content can influence access, scene unity, complexity, and provide depth cues (e.g., Gibson, 1958; Ulrich, 1977, 1983). Parsons et al. (1995) found that more biodiverse landscapes including those with rough ground cover had lower preference ratings. Such information can be the basis for a design intervention that provides suitable walking surfaces amidst a more ecologically preferable biodiverse ground cover.

Circulation Boundary Type (18) describes the edge condition of (each) identified circulation surface. Character states of this attribute vary by complexity based on the edge condition of the boundary line. In Figure 2D, 0, not relevant; 1, fragmented edge; 2, cut edge of manmade material; 3, cut edge includes additional edging of different material; 4, standard street curb; 5, manmade vertical edge (barrier) open or closed; 6, natural vertical edge (e.g., shrubs, boulders), 7, dip, gutter or ditch. Ewing and Handy (2009) found that degree of layering at the edge of streets contributed to the perception of complexity.

Skyline preferences depended on the mix of formal structural characteristics and natural content (Nasar and Terzano, 2010). Several attributes address these considerations.

Skyline Width in Frame (19) measures the distance the skyline extends across the horizontal axis of the frame; 0–100% (Figure 2E).

**Figure 3:**
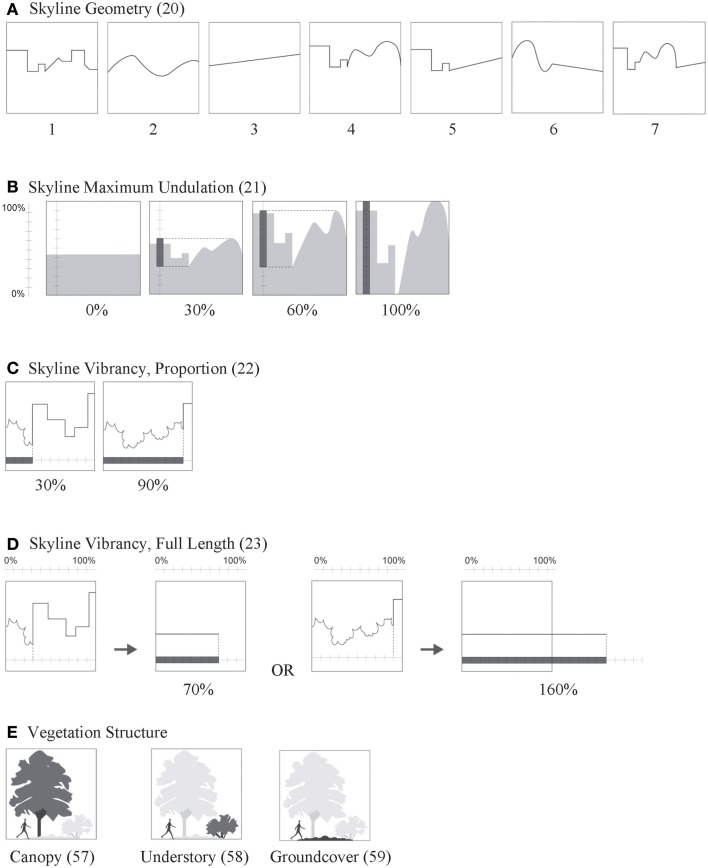
**Visualized definitions—character states of attributes with complex spatial definitions, part 3**. Attribute ID numbers given in parenthesis. Descriptions of numbered character states for attributes **(A–D)** are given in the section called Structure Attributes; for attribute **(E)** see section called Landscape Attributes.

Skyline Geometry (20) is based on the shape of the skyline regardless of content (i.e., built vs. natural objects). In Figure 3A, 0, no skyline; 1, sharp corners; 2, curves; 3, straight line; 4, sharp corners + curves; 5, sharp corners + straight lines; 6, curves + straight lines; 7, all shapes. Alexander Pattern #116 “cascade of roofs” is about the visual impact of skyline structure in urban settings.

Skyline Maximum Undulation (21) relates the maximum amount of vertical shift in the skyline. It is measured as the distance between the highest and lowest points of the skyline, and reported as % of the vertical frame height, 0–100% (Figure 3B).

Interaction between light and the surface water and/or waxes of foliage produces a sparkle effect or change in vibrancy which can be amplified in response to atmospheric changes, even small fluctuations in wind, thermal, and light conditions.

Skyline Vibrancy, Proportion (22) is the proportion of frame width occupied by the canopy–sky interface, the place where foliage vibrancy is most easily measured (Figure 3C).

Skyline Vibrancy, Full Length (23) measures the length of the canopy–sky interface along its path (i.e., includes all vertical shifts). The length is reported as a percent of the frame width and can range from 0% to infinity (Figure 3D).

#### Content attributes (Table 3)

These attributes are non-landscape objects known to be of relevance to preference or restoration responses.

Natural Phenomena (24) records the presence of engaging aspects of nature including ones that are predictable—like changes associated with the diurnal and seasonal cycles (sunsets and fall color), or unpredictable—like the occurrence of reflective water or ephemeral changes (striking cloud formations). Examples of character states are 1, light beams; 2, rainbows; 3, snowing; 4, fog; 5, visible rain; 6, continue the checklist as needed. Ryan et al. (2014) recommended the use of engaging design elements whose presence or qualities change over time thereby offering a promise of new information to come. Perceptual illusion, where apparent size or proximity of objects alters the viewer's sense of perspective, can result from looking through fog and water (Perea, 2011).

Water Form (25) records the presence of water, its source (installed or natural), its edge form (engineered or natural) and its aesthetic (stylized or natural). Character states include 0, no water; 1, installed water with engineered edges and a stylized aesthetic (e.g., fountain, pool); 2, installed water with engineered edges and a natural aesthetic (e.g., retention pond with naturalized edges); 3, natural water body with (apparently) engineered edges; 4, natural water body with (apparently) natural edges. Engineered edges refers to the use of manmade elements to contain the water. Water in all its forms has been shown as restorative (White et al., 2010). Water body presence always increases openness of a scene. Landscape reflections onto still water bring symmetry and complexity to a scene (Berdan, 2004). Alexander Pattern #64 suggests design approaches that increase water body presence in urban settings based on research about its positive effects.

Distinct Shadows (26) are scored if they bring great visual interest to a scene; No-Yes. Strong patterns appear when trees cast parallel shadows and these can produce affective responses: horizontal lines are associated with tranquility and rest, vertical lines–strength, and ascendancy, oblique or diagonal lines–movement, action and change, and curved lines–quiet, calm and sensual feelings (Berdan, 2004). Shadows also provide depth cues and their direction gives information about time of day and cardinal direction. Painters rely on the same interpretive outcome when adding shadows to convey depth realistically, making 2-D objects appear as 3-D.

Focal Objects (27) engage the viewer and guide the visual investigation of what is around. They can be an object or element that is unusual to scene content. Focal objects provide a strong sense of organization, bringing coherence to the scene. Presence is scored (No-Yes) and object type is identified. Examples include public art, a fountain or a prominent garbage bin in a pastoral scene. Alexander Pattern #126 discusses design foundations of focal objects in pleasing urban settings.

Wayfinding Objects (28) are scored when signage, public landmarks or other wayfinding objects are present; No-Yes. Information that supports wayfinding is critical to psychological well-being (Lynch, 1960).

Lighting (29) is scored when a manmade light source of any type is present; No-Yes. Street lights are the most typical. Lighting offers safety (Stamps, 2005), enhances the usefulness of wayfinding cues (Lynch, 1960), and, in the presence of foliage, can heighten engagement in ways akin to sun-foliage interactions which produce shadow casting and vibrancy effects. Alexander Pattern #252 describes the role of light in creating functional social spaces.

Seating (30) is the presence of an object designed for, or obviously used for sitting; No-Yes. Seating serves as a place to pause and collect information. If well placed, it offers safety by acting as a refuge. Abdulkarim and Nasar (2014) found that the presence of seating in an urban is associated with a restorative effect. Alexander Pattern #241 discusses why seat location/orientation is far more important than its style.

Windows (31) are visible to the viewer; No-Yes. Alexander Patterns #57 and #164 explain the positive impact of street windows on safety and neighborhood life. Windows also bring complexity and unpredictable engagement to a scene with their reflections (Berdan, 2004). Size and proportion of windows help establish the scale of a building (Ching, 2007).

Vehicles (32) include any type of motorized vehicles visible to the viewer; No-Yes. Vehicles are relevant to safety (harboring friend or foe, see Alexander Pattern #52), are engaging because movement pattern is unpredictable, and when parked along a street, act as a barrier to moving traffic (Clifton et al., 2008).

The Presence of Animals (33) and People (34) is related to safety (friend or foe), engagement, and depth cues come from proportional scaling using body size to infer distance. Animal presence also indicates naturalness. Alexander Pattern #74 argues that animal presence influences well-being on par with plants. People presence can serve as well-being indicator of a functional public social fabric. (Alexander Pattern #100). Both attributes are scored: 0, none; 1, 1; 2, 2 to 5; and 3, >5. The range and split points suited the data set used for testing. See Discussion Identifying Additional Attributes and Expanding Metrics on making place-based adjustments to metrics of these attributes.

Portrait (35) happens when a single subject dominates the scene. Common portrait subjects are 1, flower or small plant; 2, person or people; 3, animal(s); 4, other (and identify what it is); 0, not a portrait. Since, portrait subjects are often a source of fascination to the photographer (Berdan, 2004), this attribute is predicted to be informative in research where images are taken by the subjects.

#### Landscape attributes (Table 4)

**Figure 4:**
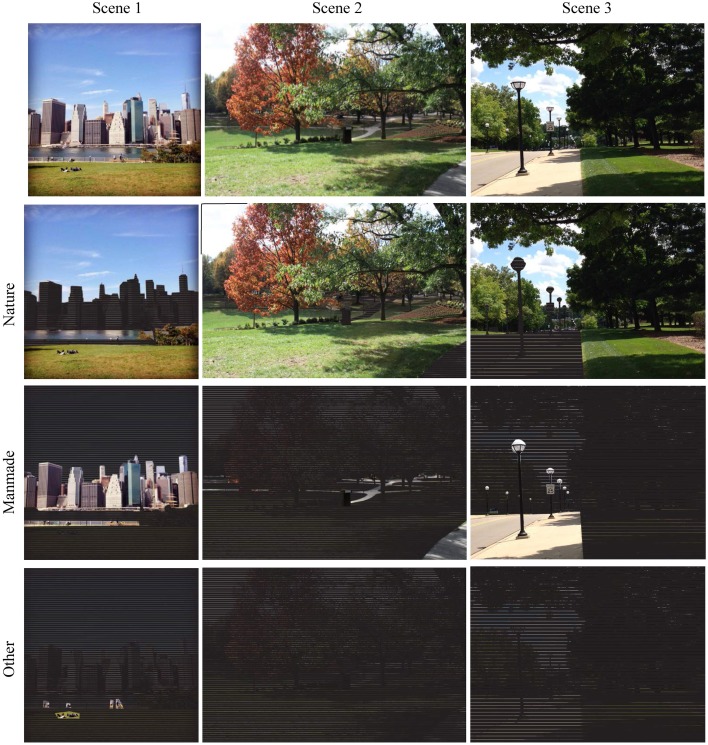
**Comparison of coverage by Nature (60), Manmade (61), and Other (62) elements for three scenes**. Attribute ID number given in parenthesis.

A hierarchy of attribute measurements starts with nature, manmade, and “other” landscape categories, the latter including humans, animals and undecipherable content (Figure 4). All landscape attributes are measured as percent of the total viewing frame area; each has a value from 0 to 100%. Nature and manmade elements are divided into subgroups, most with a demonstrated impact on preference and restoration. For nature these include Sky (Hepburn, 2010), Water (Völker and Kistemann, 2011), Vegetation (e.g., Berman et al., 2008), and Earth, the latter defined as any ground which is neither vegetated nor manmade (e.g., dirt, sand, rocks). Manmade groups are Built Structures, which include buildings and any manmade objects like sculptures or signage, and Built Ground which includes any ground surface whose materiality has been adjusted by construction such as paved roads or wooden boardwalks. Empirical work and the experience of designers supports a role for manmade structures in well-being outcomes (Alexander et al., 1977). This underlines the need to move away from dichotomizing nature and built up urban areas as the point of comparison.

**Figure 5:**
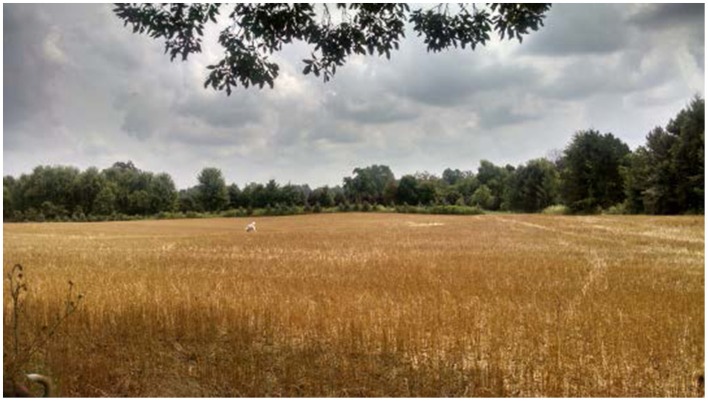
**Veiling: the superimposition of open matrix foliage on any non-plant surface**. Along the top of the viewing frame, sky is veiled.

An interesting discovery happened early our process when trying to quantify the proportion of a scene holding vegetation, a seemingly easy task. While examining images, there were many instances where open matrix vegetation (e.g., an open web of leaves surrounding an extended tree branch) partially masked what was beyond. What was beyond was generally understood, but sometimes with less certainty. We call this a *veiling effect* (e.g., Figure 5). Further investigation of images and extended field observation by the lab team made it clear that veiling is a common occurrence and often (a) heightens engagement by presentation of mystery (what lies beyond this thin veil?), (b) brings fascination via the amplified sparkle/shimmer due to the interplay foliage with light and breeze, and (c) increases depth perception because its layering effect and its production of shadows support the use of proportional scaling. Akin to a veiling effect, Kaplan et al. (1972) reported that a most preferred scene type had a well-lit clearing that was partly obscured from view by intervening foliage. The veiling effect is also akin to Alexander's pattern #238 on the value of filtered light. Recognition of the veiling effect required that landscape attributes be quantified in two ways—recognizing or ignoring veiling.

When veiling is considered, landscape elements are measured separately as veiled or open (unveiled). Veiled attributes are: Sky Veiled (36), Water Veiled (37), Earth Veiled (38), Built Structures Veiled (39), Built Ground Veiled (40), and Other Veiled (41). Veiling Vegetation (42), is the sum of all attributes and can be used to test hypotheses about the total veiling effect.

**Figure 6:**
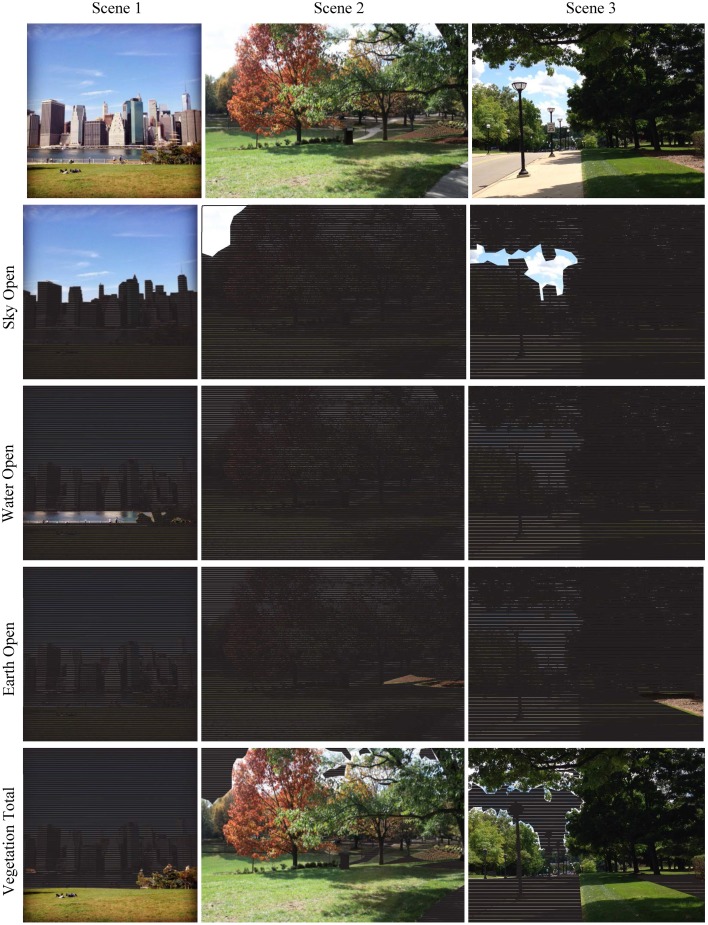
**Visual comparison of natural landscape components found in three scenes**. Nature is defined by the attributes (ID#): Sky Open (43), Water Open (44), Earth Open (45), and Vegetation Total (53) which includes all areas covered by veiling vegetation.

**Figure 7:**
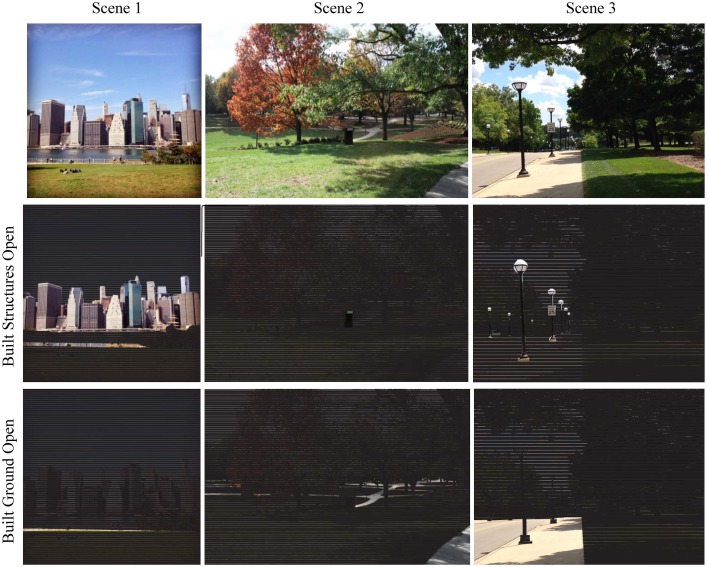
**Visual comparison of manmade landscape components found in three scenes**. Manmade is defined by attributes (ID#): Built Structures Open (46) and Built Ground Open (47).

Unveiled attributes, are not covered by any intervening foliage, include: Sky Open (43), Water Open (44), Earth Open (45) (Figure 6), Built Structures Open (46), Built Ground Open (47) (Figure 7), and Other Open (48). To account for 100% of image content, the variables above are added to Non-veiling Vegetation (52), vegetation not participating in veiling.

When veiling is ignored, each landscape group is represented as the addition of its Open and Veiled components: Sky Total (49), e.g., Sky Open + Sky Veiled; Water Total (50), Earth Total (51), Built Structures Total (54), Built Ground Total (55), Other Total (56). To account for 100% of image content, the variables above are added to Vegetation Total (53) shown in Figure 6, which is the sum of Non-veiling Vegetation (52) and all Veiling Vegetation (42).

**Figure 8:**
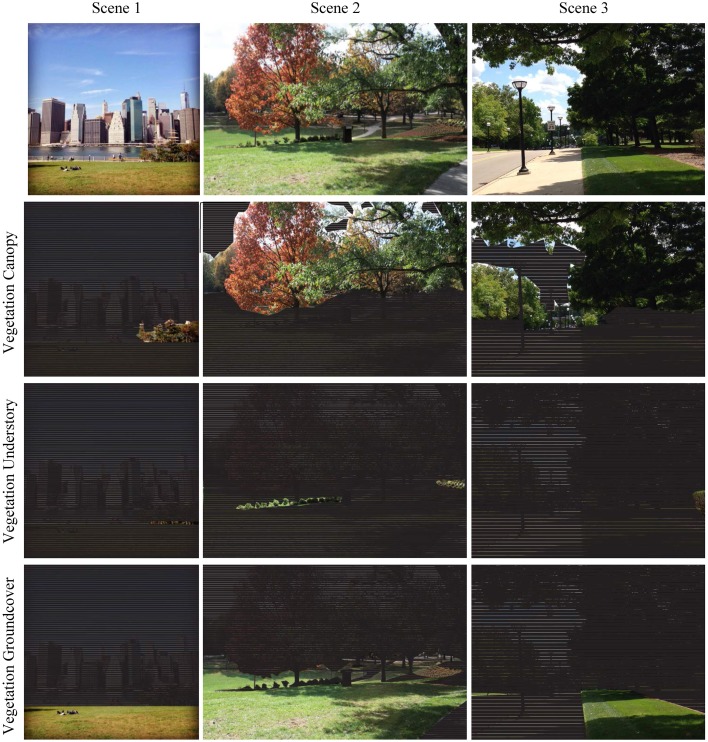
**Analysis of vegetation attributes (ID#) in terms of vertical structure for three scenes in terms of Vegetation Canopy (57), Understory (58), and Groundcover (59)**.

Vegetation is also evaluated in terms of vertical structure (Figure 8) by subdividing Vegetation Total (53) into Vegetation Canopy (57)–vegetation 8 feet or greater, Vegetation Understory (58)–plants ranging from 3 to 8 feet tall, and Vegetation Groundcover (59)–herbaceous plants or low shrubs up to 3 feet tall. Plant heights were estimated by proportional scaling, comparing the size of plants to known objects in the scene or knowledge of their size relative to human scale (Figure 3E).

The overall balance of nature and manmade elements in a scene can be assessed Nature (60), Vegetation Total + Sky Open + Water Open + Earth Open; Manmade (61), Built Structures Open + Built Ground Open; and Other (62), Other Open. Note that all veiled areas are included in Vegetation Total.

## Discussion

This research produced a systematic approach to meet the challenge of identifying which specific physical attributes of an environmental setting are most likely to influence preference and restoration responses. Physical attribute identification was the result of a triangulation process invoking environmental psychology and aesthetics theories, principles of design founded in mathematics and aesthetics, and empirical outcomes and design practices regarding the role of specific physical attributes in preference or restoration responses. The first product was identification of 10 structure-content properties predicted by theory to contribute to preference and restoration (Table 1). The second product was identification of 62 measurable physical attributes, each of which attended to one or more of the 10 theory-based properties and was linked empirically to preference or restoration (Tables 2–4).

The attributes can be used in preference-restoration research during pre-study image selection or post-study interpretation of results to enable a more specific type of scene interpretation than presently exists. Consider depth cues, one of the 10 properties of a preferred or restorative scene (Table 1). In 1950, Gibson demonstrated that depth perception is based on perceived relationships between objects and their proportional size relative to a background surface, with ground surface being the most important (Gibson, 1950). Although this has been confirmed many times since (e.g., Bian et al., 2005), depth is a variable rarely found in empirical studies of preference/restoration, most likely because it is tough to measure in 2D images [e.g., protocols in Lebreton et al. (2014) for images and Schirpke et al. (2013), for landscape metrics using GIS modeling].

However, we have identified many readily measured physical attributes that provide depth cues (Tables 2–4) with our approach. The depth cue array includes 9 structural attributes–position of the horizon line (1) and skyline (2), type of perspective (3) and scenography (4), degree of water expanse (7), position of nearby tree trunks (9), position of objects that frame a scene (10), proximity of people–for human scaling (13), and the dominant groundcover type on circulation surfaces (17). Five content attributes are the presence of distinct shadows (26), seating (30), windows (31), vehicles (32), and people (34), all of which serve as proportional scales for sizing in support of distance estimation. Landscape attributes include the total area of veiling vegetation (42) or any of its subsets (32–41). This group of attributes can be used in any combination to test hypotheses about the physical nature of depth cues that contribute to preference and restoration. The same can be said for attributes associated with the other nine structure-content properties that emerged from theory evaluation. Such research outcomes are exactly what is required for evidence-based design.

Our protocols for attribute identification ignore the debate about relative contributions of cognitive, affective, and other aspects of human response to nature such as cultural tradition and the nature of transcendence. We think these very issues, can be better investigated with a well-founded and common set of physical attributes for testing.

### Hypothesis testing

The physical attributes listed on Tables 2–4 articulate a starting point for deciphering which scene stimuli dominate or collaborate in preference and restoration responses. It was our intent to provide a manageable approach for testing hypotheses about which physical attributes of a scene are likely to intensify, neutralize, or reduce the impact on preference or restoration over a range of outdoor settings. With a diverse selection of attributes, it is possible to determine if they act singly or in concert—and if so, in what combinations. For example, data can be analyzed to determine (a) if and how cohorts of attributes contribute to preference and restoration, (b) whether there are critical points along the continuum from highly natural to highly urban where different physical attribute cohorts are most functional in generating preference and restoration, (c) the importance of the sky as a nature element in highly built areas, and (d) whether the amount of veiling in scene is related to preference and fascination.

Attributes can serve as independent variables or clusters to evaluate the physical premises of preference and restoration outcomes using standard statistical methods such as cluster and factor analyses, multiple, and logistic regression. Where data analysis identifies specific attribute clusters that bring preference and restoration, we will be closer to understanding how to design for a gestalt reaction that brings positive outcomes.

How the results in Tables 2–4 are used will depend on the question asked by the investigator. Consider the attribute Horizon Line Position (#1, Table 2). One researcher might choose to measure the contribution of horizon line position to preference or restoration responses while another might investigate which or how the 3 theory-based-properties of horizon line position are involved in preference and restoration (e.g., a viewer's sense of structural coherence in a scene, the provision of depth cues, and support for information gathering). And yet another researcher might read Tables 2–4 vertically to identify which attributes are related to a property (e.g., depth cues), then test to discover which ones make the strongest contribution to preference and restoration, and whether the result is maintained across landscape types, cultural context, age class, and so on.

### Identifying additional attributes and expanding metrics

The attribute list and its associated metrics were designed for universal application, useful for any landscape type. However, this list is not meant to be exhaustive. Place-based differences, the goal of an investigation, the evolution of theory, and the availability of new technologies will require the development of new attributes and metrics. Our research offers a protocol for identifying appropriate and measurable attributes and adding new metrics to existing ones.

While our sight was set on developing the most robust definitions, there is likely some bias because most of the images used during metrics building came from natural or moderate density urban settings in the relatively flat Midwestern U.S. Consideration of very different geographic settings, habitat types or land use could precipitate identification of more attributes owing to a greater range of structure and content than we dealt with. Karmanov and Hamel (2008) point out the paucity of research on restorative features of urbanized areas beyond those supplied by green spaces and water. New attributes for highly urbanized environments could be identified and defined with the method presented here.

Evaluation of places with different landscape structure might require new metrics for an existing attribute. For example, in a desert setting where trees do not exist, Trunk Position of Very Nearby Trees (9) could be customized by specifying tall, sturdy cacti for trees (e.g., *Yucca brevifolia*, Joshua Tree). If instant cover (safety, Table 2), is under study, this choice would be appropriate. However, if the cactus is covered with thorns (e.g., *Carnegiea gigantea*, Saguaro Cactus), interpretation of the response might be different. Both types of cacti would be good tree substitutes if the nearby “tree” is serving as a proportional scale (depth cue, Table 2). The point is that customization should recognize theory predictions that underlie an attribute. When adding or reinterpreting a character state it is important to go back to the theory basis of the attribute's selection (shown in Tables 2–4) when designing an experiment or interpreting its results.

Some attribute metrics scored as No-Yes (26–32) can be expanded to answer different questions. It would be valuable for a designer to know, for example, if the contribution of window presence (31) to preference or restoration depends on the number of windows or the percent of a viewing frame occupied by windows. Expanded metrics for window presence would be more sensitive to differences in the impact of windows in different settings such as city center, suburban residential, urban parkland, and wildlands. Such information would fortify the evidence-base for well-being designers who can use the information to choose vantage points or create screens to keep the amount of visible window within an optimal range.

Other place-based adjustments to attributes include those scored by frequency class. Adjustment of range and split points should attend to the range of response found in a data set or the need for greater specificity owing to the hypothesis being tested. Only 2 of the 62 attributes presented here are eligible for this type of adjustment—Animal Presence (33) and People Presence (34). The range and split points for both are: 0, none; 1, 1; 2, 2 to 5; 3, >5 because a focus on low counts had the greatest discriminatory power with the data set used. However, data sets with scenes from city centers or recreational areas might benefit from a larger range with more split points. Simple counting is one such adjustment.

The insights from design theory and practice were indispensable as we identified attribute candidates and developed metrics. They also suggest which adjustments to the building blocks of environmental structure (like horizon line position or sky: manmade proportions in a scene) are likely to impact the viewer's sense of spatial structure. Consequently, we strongly recommend that research teams include both scientist and designer for work on attribute development, translational application, and evaluation of evidence-based designs that use such research outcomes.

### The challenge of defining less measurable attributes

Some design principles involved with naturalness are less tractable because analysis methods are time consuming or require expertise or available computer algorithms. Evaluation of structural proportion and fractal geometries are in this group. Color, a popular focus of human-nature interaction studies, is complex to evaluate, even when the focus is on vegetation (which is often not green; what is green anyway?). In fact, the color of nature is highly variable. Computer algorithms for measuring color are excellent, their limitation coming only from the color trueness of the image under evaluation (Nishiyama et al., 2011). The field of visual aesthetics has made significant headway in its methodology for evaluating human preference in terms of color and spatial structure (Palmer et al., 2013), as well as complexity, symmetry, line orientation, spatial proportion, compositional balance, and the role of meaningful objects in biasing compositional sense. For example, Berman et al. (2014) found that degree of perceived naturalness was highly correlated with low level visual features defined by average color saturation and hue diversity, as well as the density of contrast changes and straight lines.

### Why measuring attributes improves image selection and interpretation

Most importantly, the approach we present supports image selection along a more finely grained continuum from natural to manmade, the range of environments encountered in everyday life. This is of particular value for tests about the role of personal experience or cultural norms in response to nature, where the standard dichotomous choice (natural vs. urban) is not sufficient. We suspect that a more finely-grained natural-urban scale is needed to locate optimal restoration points for an upstate New York suburban community compared to the one living in Manhattan.

The choice of images for testing will also improve by using multiple criteria to identify incorrect assumptions (e.g., when tree presence does not have a positive effect) and to detect potentially confounding effects (e.g., why preference or restoration response to water is highly variable). Practically speaking, attribute scoring of an image offers a guided examination that uncovers often unrecognized cues involving depth perception, human scaling, unnoticed content, etc.

### Using research on physical attributes to provide an evidence-base for design

Landscape architects, architects, and planners shape and form the spaces that humans use, giving them great restorative agency in the world. For the best outcome, well-being designers require information about which physical attributes of nature—spatial structure and content, are most likely to support health, especially in urban settings. Research outcomes about the contribution of physical attributes to preference and restoration (some already known) can be used to guide design professionals aiming to join aesthetics, ecological, and psychological principles for the production of restorative urban spaces. Here, are a few examples of how evidence-base design could translate research outcomes. (1) Research outcome: the presence of framing foliage triples preference/restoration ratings of the urban scene holding < 20% total vegetation; design solution: in densely built areas, locate trees/ plants with appropriate architecture so that favored stopping places include nearby foliage in their viewshed. (2) Research outcome: as the length of skyline vibrancy increases so does preference/restoration; design solution: maximize the opportunity for distant views with skylines dominated by tree canopies (a view which is borrowed and free) and minimize views of manmade skylines by masking or redirecting attention from them. (3) Research outcome: preference/restoration drops by half when horizon line position is not discernable from a seating area in a public park; design solution: provide other kinds of location cues, adjust the view so the horizon line is visible, or if possible, relocate the benches for a view including horizon line.

The development of a better evidence base for well-being designers will provide a realistic premise for urban greening that extends far beyond the broad generalizations currently available–like provide 10–20% natural areas and be sure that water in some form is encountered within a 1 mile radius of any residence. As urban density increases worldwide, so does the domination by built objects and the loss of ecosystem services including psychological ones (Irvine et al., 2010). There is the need to learn more specifically about the type of nature that best supports well-being (Bratman et al., 2012; Shanahan et al., 2015). In addressing this question, our research is offers a route to find these answers. The knowledge can be applied at all scales from bench placement and streetscape design, to large-scale city park design and selection of path systems in wild places.

### Conflict of interest statement

The authors declare that the research was conducted in the absence of any commercial or financial relationships that could be construed as a potential conflict of interest.
